# Therapy with Ulipristal Acetate in a Hypertensive Patient

**DOI:** 10.1155/2018/1091520

**Published:** 2018-11-01

**Authors:** A. Alcalde Dominguez, J. Rabasa Antonijuan, M. Cusidó Gimferrer, M. Jiménez Ortuño

**Affiliations:** ^1^Servicio de Obstetricia y Ginecología, Hospital Universitari Sagrat Cor, Barcelona, Spain; ^2^Servicio de Obstetricia y Ginecología, Centro Médico Teknon, Barcelona, Spain

## Abstract

Ulipristal acetate (UPA) is a medical therapy for patients with symptomatic uterine fibroids. The drug has shown efficacy in the control of heavy menstrual bleeding and, as a consequence, in anaemia improvement. We report the case of a hypertensive patient treated with two courses of UPA. In addition to its observed benefits on hypermenorrhea caused by uterine fibroids, no exacerbation of the underlying disease was observed. No adverse effects were observed, and blood pressure levels were well controlled throughout.

## 1. Introduction and Objectives

Uterine fibroids are benign tumours of the smooth muscle of the uterus [1]. They are so common, with a 20–25% prevalence rate in women of reproductive age. These can be asymptomatic, but in up to 40% of cases, they can be associated to abnormal uterine bleeding, pelvic pain, infertility, and/or voiding symptoms, depending on their location and size [2].

Symptomatic fibroids can be treated either surgically or pharmacologically. The therapeutic approach will depend on several factors, such as the patient's signs and symptoms, age, and reproductive plans. In general, medical therapy will be prioritized when the primary symptoms are bleeding and hypermenorrhea causing anaemia. Surgical treatment will be chosen if the associated symptoms are due to compression of adjacent organs or infertility [3].

Ulipristal acetate (UPA) is a medical therapeutic option for the treatment of symptomatic fibroids. UPA is a steroidal compound that belongs to the class of selective progesterone receptor modulators (SPRM), which exert a tissue selective agonist, antagonist, or mixed agonist-antagonist activity in target cells. UPA is indicated both for repeated-intermittent and preoperative treatment of moderate to severe symptoms of uterine fibroids in adult women of reproductive age.

We report the case of a hypertensive patient treated with UPA. We aim to find out, in addition to its beneficial effect in the control of symptoms caused by uterine fibroids, whether UPA may influence the patient's underlying hypertension.

## 2. Case Report

A 46-year-old patient (weight: 70 kg; height: 161 cm) with a history of hypertension treated with losartan 50 mg/day and hydrochlorothiazide 12.5 mg/day came to the gynaecology clinics due to menometrorrhagia. A transvaginal ultrasound showed a 60 × 37 × 42 mm anteflexion uterus with a type I (according to the FIGO classification) submucous fibroid measuring 39 × 33 × 32 mm and a smaller intramural fibroid of 21 × 17 × 16 mm, both in the posterior uterine wall. A surgical hysteroscopy was scheduled. Preoperative blood testing showed an Hb value of 10.1 g/dl.

The surgical hysteroscopy procedure evidenced a type I submucous fibroid (according to the FIGO classification) measuring 3.5 cm approximately, and resulted in the removal of 3/4 of it.

**Figure 1:**
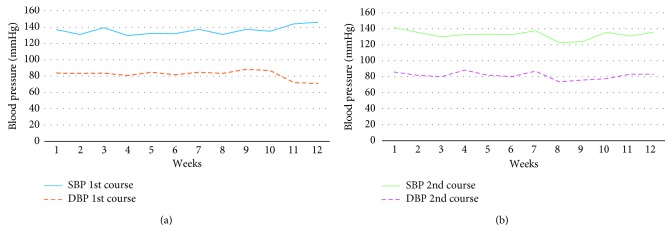
Systolic and diastolic blood pressure follow-up during the two courses of UPA treatment: (a) STP/DBP follow-up during 1st course with UPA; (b) STP/DBP follow-up during 2nd course with UPA.

The patient attended the physician office one month after surgery reporting the persistence of hypermenorrhea. A type II submucous fibroid measuring 2 cm in the posterior uterine wall was found by transvaginal ultrasound, and reported Hb value was 10.2 g/dl. She was offered and accepted to undergo a 3-month treatment course of UPA. During the treatment period, a blood pressure (BP) weekly control was performed to the patient, as instructed. The controls revealed a mean systolic BP of 136 mmHg and a mean diastolic BP of 86 mmHg, similar to the recorded BP figures before initiation of UPA therapy.  Figure 1 shows the BP progress throughout the 3-month treatment course with UPA. After this treatment period, the persistence of the fibroid was reported, and Hb value increased up to 12.8 g/dl.

The patient decided to follow a wait-and-see approach. Two months later, hypermenorrhea symptoms reappeared, and a decision was made on starting a new UPA treatment course. During this second UPA treatment course, BP values were again within the normal range (mean systolic BP = 133 mmHg; mean diastolic BP = 82 mmHg) (Figure 1), and no adjustments of antihypertensive therapy were needed. Three months after the second treatment course, the patient's Hb value was 12 g/dl and symptoms of hypermenorrhea had decreased.

During the treatment with UPA, no clinical events were reported, nor any sign of drug interactions. The antihypertensive agent dose remained unaltered.

## 3. Discussion

The present clinical case reports the use of UPA in a hypertensive patient on treatment with antihypertensive agents, showing its efficacy on the control of symptoms due to uterine fibroids without negative impact on blood pressure values, nor interactions among drugs.

In the context of the medical treatment of uterine fibroids, there is no consistent evidence supporting the efficacy of combined hormonal contraceptives, which, in addition, have no indication for this condition according to its Summary of Product Characteristics (SMP) [4]. On the contrary, GnRH analogues efficacy in bleeding control and volume reduction has been demonstrated, but their benefits are limited to a maximum 6-month therapy due to its secondary effects related with hypoestrogenic status [5, 6].

UPA is a first-line therapy for patients with symptomatic uterine fibroids. Its efficacy in the control of uterine bleeding associated to fibroids, as well as in the reduction of their size, has been demonstrated in different clinical studies [7–10]. Its safety profile has also been widely shown [7–10]. Specifically, results from study PEARL III ext. II showed that blood pressure (BP) figures were not significantly altered throughout the eight UPA treatment courses [11].

In a recent randomized placebo-controlled 3-arm parallel clinical trial, Simon et al. [12] compared the efficacy and tolerability of a 3-month course of 5 mg and 10 mg of UPA or placebo. Among the 133 patients who completed the follow-up period, the most common adverse events (>5%) were hot flushes, blood creatinine phosphokinase elevation, and hypertension. Specifically, 5 of 6 hypertension events reported were a worsening of pre-existing disease at baseline, and the other case was diagnosed during the follow-up period. Nevertheless, none of the cases of hypertension was considered related to treatment. Indeed, the majority of these patients were obese and not treated for hypertension at baseline, which may contribute to explain these cases. Of note, hypertension adverse events were not referred in other clinical trials with larger sample size and in studies of repeated courses [7–9, 11].

Recently, the European Medicines Agency has carried out a safety assessment on UPA related to four cases of hepatic failure leading to transplantation. The final conclusion has been that causality on UPA cannot be attributed nor ruled out and that its benefit-risk balance remained favourable. The drug can be prescribed as liver tests rule out any previous underlying hepatic disease. No hypertension adverse event has been observed related to this assessment.

## 4. Conclusion

To conclude, we report a case in which UPA therapy was successfully used in a premenopausal patient with menorrhagia caused by uterine fibroids and hypertension. No negative impact on blood pressure levels nor interactions with antihypertensive agents were observed. Further studies are needed to strengthen the safety of UPA in hypertensive patients with symptomatic fibroids.
